# Investigation of Lake Hévíz Mineral Water Balneotherapy and Hévíz Mud Treatment in Murine Osteoarthritis and Rheumatoid Arthritis Models

**DOI:** 10.1155/2018/4816905

**Published:** 2018-08-27

**Authors:** V. Tékus, É. Borbély, T. Kiss, A. Perkecz, Á. Kemény, J. Horváth, A. Kvarda, E. Pintér

**Affiliations:** ^1^Department of Pharmacology and Pharmacotherapy, Medical School, University of Pécs, H-7624, Pécs, Szigeti U. 12, Hungary; ^2^János Szentágothai Research Centre, University of Pécs, H-7634, Pécs, Ifjúság U. 34, Hungary; ^3^Saint Andrew Hospital for Rheumatic Diseases, H-8380, Héviz, Dr. Schulhof Vilmos Sétány 1, Hungary; ^4^PharmInVivo Ltd, H-7629, Pécs, Szondi György U. 10, Hungary

## Abstract

Arthritic diseases are the most frequent causes of chronic pain and disability. Rheumatoid arthritis (RA) is an autoimmune disease characterized by synovial inflammation and progressive structural joint damage. Osteoarthritis is a degenerative process of the articular cartilage associated with hypertrophic changes in the bone. The aim of the present study was to investigate the anti-inflammatory and analgesic effects of Hévíz thermal water and mud in monosodium iodoacetate- (MIA-) (25 mg/ml, 20 *μ*l i.a.) induced osteoarthritis and Complete Freund's adjuvant- (CFA-) (1 mg/ml, 50–50 *μ*l s.c) induced rheumatoid arthritis murine models. The mechanonociceptive threshold of female NMRI mice (n=6– 8 mice/ group) was measured by aesthesiometry, and paw volume was monitored with plethysmometry, knee joint diameter with digital micrometer, and dynamic weight bearing on the hind limbs with a Bioseb instrument. Periarticular bone destruction was assessed by SkyScan 1176 in vivo micro-CT. Inflammatory cytokines were detected by ELISA in plasma samples. Treatments (30 min, every working day) with tap water, sand, and a combined therapy of tap water and sand served as controls. Hévíz medicinal water and combined treatment with water and mud significantly decreased the mechanical hyperalgesia and knee oedema in MIA-induced osteoarthritis model. However, balneotherapy did not influence mechanical hyperalgesia, weight bearing, or oedema formation induced by CFA. Neither medicinal water nor mud treatment ameliorated deep structural damage of the bones or the joints in the animal models. On the basis of the present findings, we conclude that balneotherapy is an effective complementary treatment to reduce the pain sensation and swelling in degenerative joint diseases such as osteoarthritis. Our experimental data are in agreement with the previous human studies that also confirmed antinociceptive and anti-inflammatory effects of thermal water and Hévíz mud treatments.

## 1. Introduction

Balneotherapy (BT) traditionally means bathing in mineral and/or thermal waters from natural springs; besides them treatments with gas molecules (e.g., CO_2_, H_2_S), peloids (mud) and other natural compounds (e.g., hay) can be listed into the balneological interventions [1]. The bottom of the largest European thermal lake named Hévíz is covered by a special mud, which can be useful for medical prospects [2]. The spring of the lake originates from 38 m deep cave, and the bed of the lake is covered with 6-8 m thick mud layer, mainly organic Hévíz mud, which is dark-grey in colour and soft in texture. The water of Lake Hévíz contains solid and gaseous compounds therefore its composition joins the beneficial forces of carbonate, sulfur, calcium, magnesium, and hydrogen carbonate-containing waters [3]. The thermal water and mud were formed in the ancient Pannonic-sea tens and they are suitable for complex physiotherapy treatments. The effective substances of the Hévíz mud were described previously [4–6]. BT is widely used to amplify the effects of drug treatments and recommended for a broad spectrum of diseases, such as arthritis, dermatitis, and fibromyalgia [7–9]. The efficacy of BT for osteoarthritis has been approved by several randomized clinical trials but beneficial actions of this treatment are often manifested in subjective improvements of the pain sensation and the quality of life [1, 10].

Osteoarthritis is a common chronic degenerative disorder associated with pain and debilitated state with a progressive damage of articular cartilage, moderate inflammatory changes in the tissues in and around the joints, and in severe cases formation of osteophytes and bone cysts [11]. The mode of action by which BT reduces the signs of OA and other broad spectrum rheumatologic disorders is not elucidated yet. The beneficial action is probably due to the combined mechanical, thermo, and chemical effects of mineral water and mud.

The exact molecular mechanism of balneotherapy is still not fully understood; however its efficacy was confirmed by several human studies [12, 13]. Tap water controlled double blind balneotherapy clinical trials were reported in rheumatoid arthritis (RA) or osteoarthritis (OA) patients in Hungary [2, 3, 6, 10, 14, 15]. The principal aim of the present study was to develop and validate available experimental animal models of osteo- and rheumatoid arthritis for investigation of the potential anti-inflammatory and analgesic effects of Hévíz thermal water and mud. Although human studies and meta-analyses presented clear evidence providing the efficacy of BT, well conducted, controlled animal studies are still imperfect in the balneological research. Therefore our methodology could be essential to elucidating the mechanism of the therapeutic impact of BT.

## 2. Materials and Methods

### 2.1. Animals

Experiments were performed on 12–16-week-old female NMRI mice. All animals weighing 45-50 g were kept in the Animal House of the Department of Pharmacology and Pharmacotherapy of the University of Pécs at 24-25°C and provided with standard chow food and water* ad libitum *under 12-hour light/dark circle. Mice were housed in groups of 5–10 in polycarbonate cages (330 cm^2^ floor space, 12 cm height) covered by wood shavings bedding. The total number of mice used in the study was 92, and they were allocated to experimental groups with randomized section. To attenuate the number of the experimental animals, the same mice were used for the functional measurements and imaging techniques, for the cytokine measurements as well as for the histopathological evaluation.

The investigators were always blinded to all the treatment. Before functional experiments two measurements were fulfilled for habituation purposes and these results were not considered for the final analysis.

### 2.2. Ethics

All experiments were executed according to the 1998/XXVIII Act of the Hungarian Parliament on Animal Protection, Consideration Decree of Scientific Procedures of Animal Experiments (243/1988) and Directive 2010/63/EU of the European Parliament. The experimental procedures were approved by the Ethical Committee of the University of Pécs, and licence was given (licence No.: BA 02/2000–2/2012). The number of animals used was kept to the minimum necessary for the purpose of the investigation and measures were taken to avoid unnecessary distress of the animals.

### 2.3. Experimental Design

#### 2.3.1. Balneotherapy

Balneotherapy was started one week before the induction of osteoarthritis or rheumatoid arthritis and was applied for 30 min every working day until the end of the study. Mice were bathed in 34-35°C Hévíz water (freshly obtained from the spring of Hévíz, which contains among others sodium: 21.7mg/l, potassium: 6.45mg/l, magnesium: 36.1 mg/l, calcium: 82.1, hydrogen carbonate: 384 mg/l, sulphate: 63.0 mg/l, and hydrogen sulphide: 0.1mg/l and has pH 7.14 and hardness 198 CaO mg/l). Tap water-treated group served as control.

#### 2.3.2. Mud Therapy

Topical administration of Hévíz mud was designed with the help of enclosing the entire body of the animal in a transparent plastic tube leaving only the tails outside. Before closing, the tube was filled up with Hévíz mud or sand to cover the lower half of the body. Small holes were prepared in the whole of the tube providing appropriate ventilation of the animals.

#### 2.3.3. Treatment Groups of the Animals

Treatment groups were as follows: Group 1: Hévíz mineral water, Group 2: tap water, Group 3: Hévíz mud, Group 4: sand, Group 5: Hévíz water + mud, and Group 6: tap water + sand.

### 2.4. Experimental Models

#### 2.4.1. MIA-Induced Osteoarthritis Model

**Figure 1:**
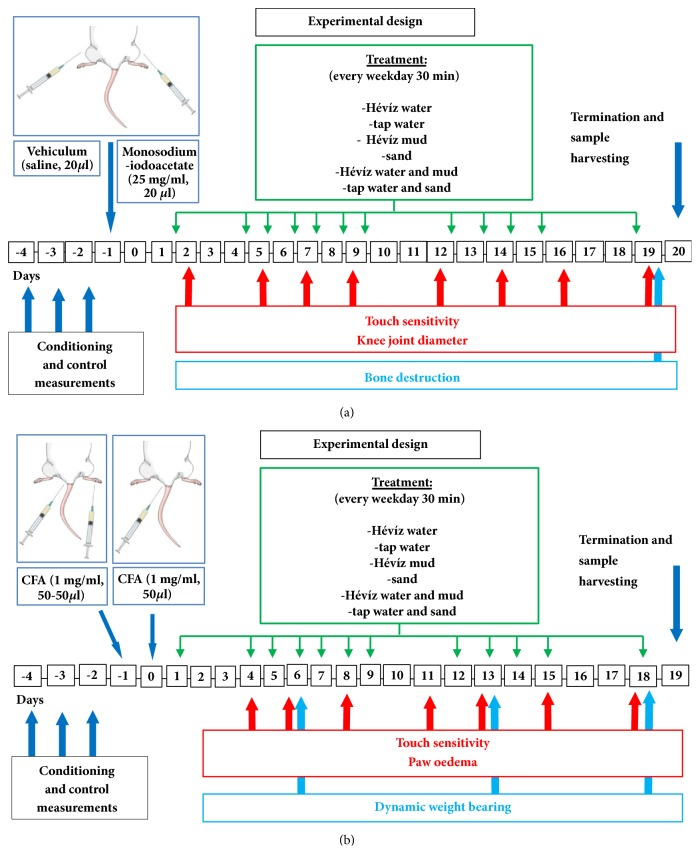
Schematic draw of protocols. (a) Osteoarthritis model and (b) Rheumatoid arthritis.

Osteoarthritis was evoked by injecting 20 *μ*l, 25 mg/ml monosodium iodoacetate (MIA, Sigma-Aldrich, St. Louis, MO, USA) dissolved in 0.9 % saline into the cavity of left knee joint cavity through the patellar ligament [16]. Investigation was performed throughout the 20-day period [17]. Mechanical hyperalgesia, knee joint swelling, and bone destruction were observed in vivo during the 3-week experimental period (Figure 1(a)), while histopathological scoring was assessed using knee joints excised at the end of the study, as described below.

#### 2.4.2. CFA-Induced Rheumatoid Arthritis Model

Complete Freund's adjuvant (CFA) containing heat-killed Mycobacterium tuberculosis suspended in paraffin oil (1 mg/ml; Sigma-Aldrich, St. Louis, MO, USA) evokes macrophage-mediated immune response. Fifty *μ*l of CFA was injected intraplantarly into the left paw and subcutaneously (s.c.) into the root of the tail. Boosting injection was applied into the tail again after one day to potentiate the systemic effects mimicking the human disease [18]. The treatment with mineral water and mud started one week prior to CFA injections and then continued for 19 days. Mechanical hyperalgesia, ankle oedema, and dynamic weight bearing were measured in vivo during the 19-day experimental period (Figure 1(b)), and histopathological analysis was executed from the tibiotarsal joints excised at the end of the study.

### 2.5. Investigational Techniques

#### 2.5.1. Measurement of Touch Sensitivity on the Hind Paw

The mechanical touch sensitivity thresholds of the plantar surface of the paws were measured with dynamic plantar aesthesiometry (Ugo Basile Aesthesiometer 37400, Comerio, Italy). This device is an automated von Frey and the most appropriate technique to study mechanical hyperalgesia in mice [18–20]. The mice can move freely; a straight metal filament touches the animal's paw and exerts an increasing upward force (maximum force of 10 g with 4-second latency) until the animal retracts its paw. The mechanonociceptive threshold was shown in grams digitally on a screen, and mechanical hyperalgesia was expressed as percentage of initial control value. Three control measurements were performed on both hindpaws for each group in the week before the experiments.

#### 2.5.2. Measurements of Paw Oedema

Paw volumes were measured by plethysmometry (Ugo Basile Plethysmometer, 7140, Comerio, Italy) prior to CFA injections, and 4, 6, 8, 11, 13, 15, and 18 days after CFA administration. Oedema was expressed in percentage compared to the initial control values [21].

#### 2.5.3. Measurement of Knee Joint Diameter

The mediolateral and the anterior-posterior diameters of the knee joint were measured with a digital micrometer (Mitutoyo, Japan) prior to MIA administration and on days 2, 5, 7, 9, 12, 14, 16, and 19.

#### 2.5.4. Measurement of Dynamic Weight Bearing

Dynamic weight bearing on the hind limbs was determined with Bioseb weight bearing instrument (Bioseb, France). This is an operator-independent system for spontaneous pain measurement and an advanced alternative for incapacitance tester. The instrument is composed of a cage, where the animals can move freely, while the special sensing floor detects the weight for each paw during the 5-min experimental period. It is combined with a video-camera and software, which records the movements of the animal and analyses the data [22]. Results were expressed as the percentage of weight distributed on the injured hind limb ([weight bearing of the treated paw /(weight bearing of the treated paw + weight bearing of the untreated paw)] x 100). After controls to establish a baseline, measurements were performed on days 6, 13, and 18.

### 2.6. In Vivo Micro-Computed Tomography (Micro-CT) Analysis of the Periarticular Bone Structure

Evaluation of periarticular bone destruction was assessed by SkyScan 1176 in vivo micro-CT (Bruker, Kontich, Belgium) with 17.5 lm voxel size at the end of the study [23–25]. Determining the changes of bone structure, standard size areas of interest (ROIs) were applied periarticularly in regions of tibia and femur and were calculated using CT Analyser® [26]. Bone volume (BV) was expressed in cubic micrometer (*μ*m^3^) and then was presented as a percentage of changes of the total volume (TV) of ROIs.

### 2.7. Determination of Plasma Cytokine Concentrations

After all functional testing had been completed on day 20 in osteoarthritis or on day 19 in rheumatoid arthritis model, all animals were deeply anaesthetized with ketamine (100 mg/kg, i.p., Richter Gedeon Plc., Hungary) and xylazine (10 mg/kg, i.m., Lavet Ltd., Hungary); then the thoracic cavity was opened and 0.5–1 ml blood was collected from the left ventricle with a heparinized syringe into ice-cold polypropylene tubes containing EDTA (40 *μ*l) and trasylol (20 *μ*l). The collected blood was immediately centrifuged (1,000 g, 20 min, 4°C), and the resulting plasma was stored at -80°C. The concentrations of the inflammatory cytokines IL-1*β* and TNF-*α* were measured by ELISA using BD OptEIA Mouse IL-1*β* ELISA set and BD OptEIA Mouse TNF (Mono/Mono) ELISA Set (BD Biosciences, USA). Detection was performed by using Labsystem Multiscan RC plate reader (LabX Ltd., Canada).

### 2.8. Histological Processing and Assessment of Joint Inflammation

Mice were anaesthetized as mentioned above, sacrificed by cervical dislocation on day 20 after MIA or 19 after CFA administration and knee joints or the whole paws (including tibiotarsal joints) were excised, respectively. After formaldehyde fixation, decalcification, and dehydration the knee joint or paw samples were embedded in paraffin, sectioned (3-4 *μ*m), and stained with Safranin O [25], respectively.

CFA-induced arthritic alterations were scored focusing on (1) infiltration of areolar tissue by mononuclear cells and synovial hyperplasia, (2) the number of leukocytes observed in the synovial tissue, and (3) cartilage destruction using a grading scale of 0 (normal) to 3 (maximal severity) for each parameter, to create composite arthritis scores [18, 27].

MIA-induced histological damage was characterized by an expert blinded to the study, with a modified Mankin semiquantitative scoring system and additional parameters. The Mankin score evaluates structure (0–6), cellularity (0–4), matrix staining (0–4), and tidemark integrity (0–1). Furthermore, synovial thickness and cellular infiltration on a scale of 0 to 3 and the osteophyte formation (0–1) were scored [28]. Mean scores were established from the sections of different animals, and composite score was calculated from these values.

### 2.9. Statistical Analysis

Data are expressed as means ± standard errors of means (SEM) of n=6–8 mice per group. Repeated measures of two-way analysis of variance (ANOVA) followed by Bonferroni's multiple comparison test was used for statistical analysis in cases of mechanical hyperalgesia, paw oedema, and knee diameter changes. Semiquantitative histopathological scoring was assessed with Kruskal-Wallis test followed by Dunn's posttest and one-way ANOVA followed by Bonferroni's multiple comparison posttest was used to compare plasma cytokine levels. Evaluations were carried out with the help of GraphPad Prism 5.01 software and P<0.05 was considered to be statistically significant.

## 3. Results

### 3.1. Hévíz Water Significantly Decreases the MIA-Induced, but Not the CFA-Induced, Mechanical Hyperalgesia of the Mouse Paw

**Figure 2:**
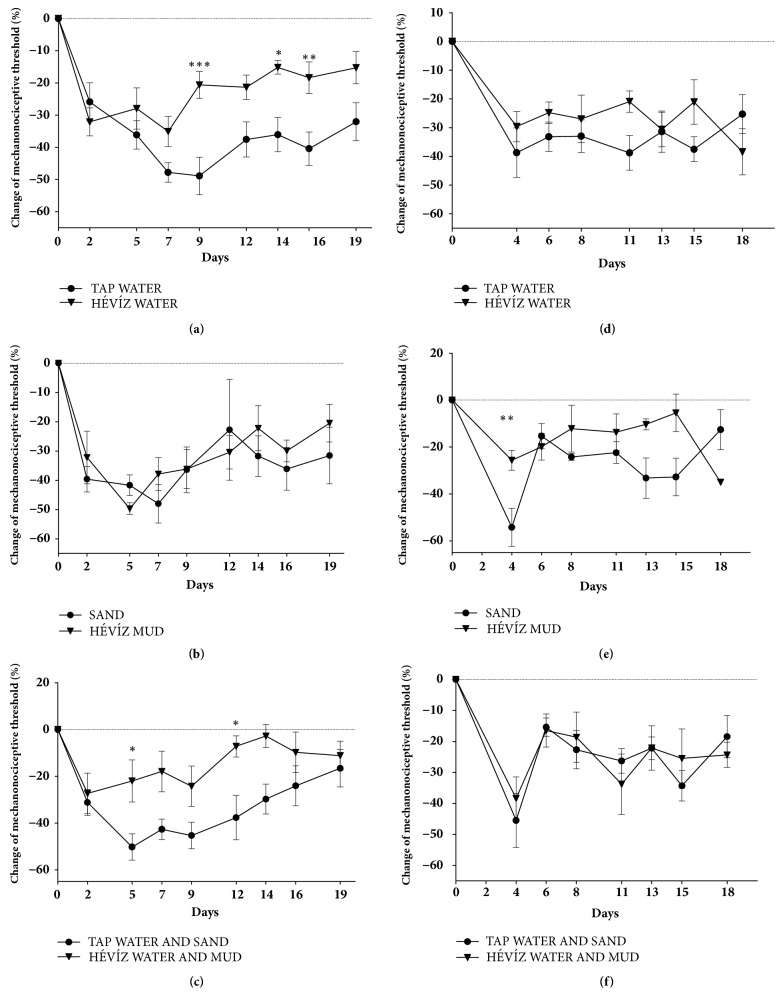
**Effects of balneotherapy on mechanical hyperalgesia in MIA-induced osteoarthritis (a, b, c) and CFA-induced rheumatoid arthritis (d, e, f) models**. Data points represent the percentage changes of the mechanonociceptive threshold throughout the 3-week experimental periods in mice treated with Hévíz water (**a, d**), Hévíz mud (**b, e**), and combined therapy with Hévíz water and mud (**c, f**) (30 mins on every weekday by the end of the study), n = 6–8/arthritic groups; *∗*p < 0.05, *∗∗*p < 0.01, *∗∗∗*p < 0.001 versus respective controls; two-way ANOVA followed by Bonferroni's multiple comparison test.

Intraarticularly administered MIA (20 *μ*l, 25 mg/ml i.a.) induced an approximately 30-40% mechanical hyperalgesia after 2 days in all groups (Figures 2(a), 2(b), and 2(c)). This mechanical hyperalgesia was gradually decreased in tap water-treated group (from 8.78 ± 0.31 g to 4.53 ± 0.59 g) and from day 9 it was significantly higher compared to the Hévíz water-treated group (-48.90 ± 5.82 versus -20.60 ± 4.10). This difference was maintained at the end of the experiment but was statistically significant on days 14 and 16 (Figure 2(a)). Significant differences of MIA-induced mechanical hyperalgesia were not developed in the Hévíz mud-treated group compared to the sand-treated control group (Figure 2(b)). But the MIA-induced robust drop of mechanonociceptive thresholds was significantly attenuated by the combination of the two types of treatment (Hévíz water and mud) compared to the respective control group (-22.02 ± 9.01 versus -45.35 ± 5.71 on D5, -7.19 ± 4.53 versus -37.71 ± 9.46 on D12, respectively, Figure 2(c)).

Four days following CFA injection approximately 30-40% mechanical hyperalgesia was developed in the tap water-treated group. Pain sensation did not differ statistically in the Hévíz water-treated group during the experiment (Figure 2(d)). In the sand-treated mice approximately 50-55% mechanical hyperalgesia developed 4 days after the CFA injection which was significantly attenuated by the daily repeated Hévíz mud treatment (-25.64 ± 4.22 versus-54.24 ± 8.13) on day 4 (Figure 2(e)). The combined therapy did not change the developed mechanical hyperalgesia in any time point (Figure 2(f)).

### 3.2. Hévíz Water Significantly Decreases the MIA-Induced Knee Swelling, but Not the CFA-Induced Paw Oedema

**Figure 3:**
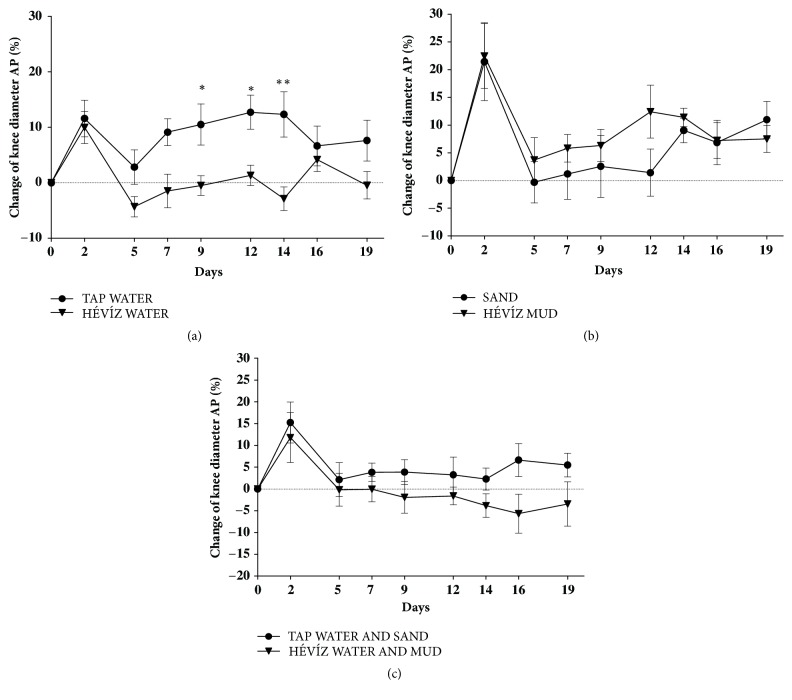
**Effects of balneotherapy on knee diameter in MIA-induced osteoarthritis model**. Data points represent the percentage changes of the knee diameter throughout the 3-week experimental periods in mice treated with Hévíz water (a), Hévíz mud (b), and combined therapy with Hévíz water and mud (c) (30 mins on every weekday by the end of the study). Data are shown as means+SEM of n = 6– 8 mice/ group; *∗*p < 0.05, *∗∗*p < 0.01 versus respective controls; two-way ANOVA followed by Bonferroni's multiple comparison test.

2 days after the MIA injection an approximately 10-20% knee diameter (AP) elevation was developed in all treated groups (Figures 3(a), 3(b), and 3(c)). Oedema formation was significantly attenuated from day 9 to the end of the experiment in the Hévíz water-treated group compared to the control tap water-treated group (Figure 3(a)). Treatment with Hévíz mud or combined treatment did not influence the change of the knee diameter compared to their respective controls (Figures 3(b) and 3(c)).

**Figure 4:**
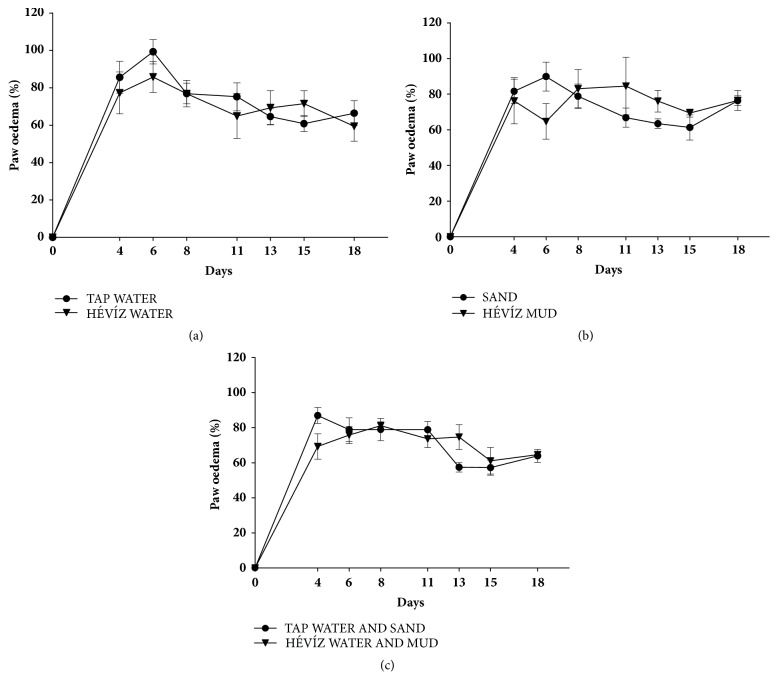
**Effects of balneotherapy on paw volume in CFA-induced rheumatoid arthritis model**. Data points represent the percentage changes of the paw volume throughout the 3-week experimental periods in mice treated with Hévíz water (a), Hévíz mud (b), and combined therapy with Hévíz water and mud (c) (30 mins on every weekday by the end of the study). Data are shown as means+SEM; two-way ANOVA followed by Bonferroni's multiple comparison test.

Approximately 70-85% hind paw oedema was developed 4 days after CFA injection in all treated groups (Figure 4(a)). None of the treatments influenced the development oedema formation during the experiment (Figures 4(b) and 4(c)).

### 3.3. Balneological Treatments Do Not Influence CFA-Induced Spontaneous Weight Distribution of the Injured Hindpaw

**Figure 5:**
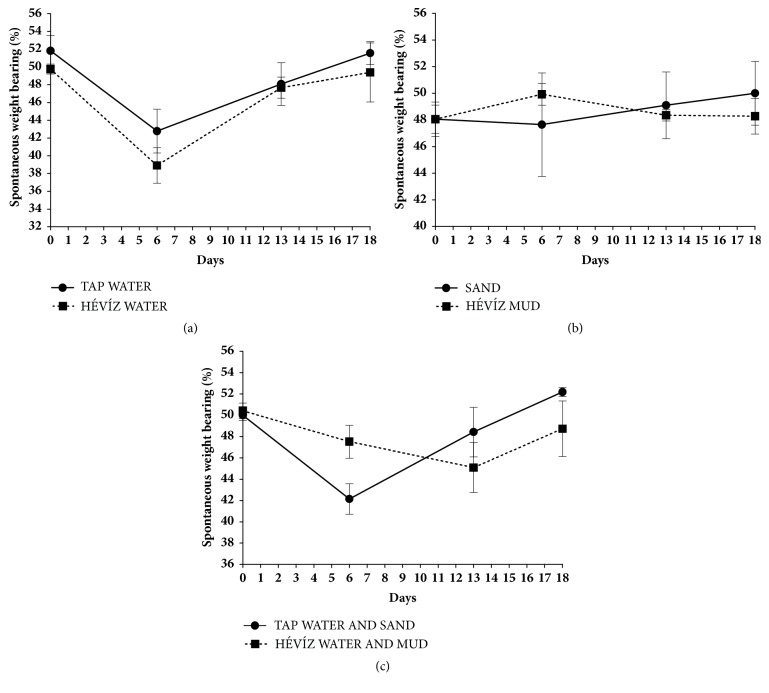
**Effects of balneotherapy on spontaneous weight bearing in CFA-induced rheumatoid arthritis model**. Data points represent the percentage changes of weight bearing of the injured hind paw throughout the 3-week experimental periods in mice treated with Hévíz water (**a**), Hévíz mud (**b**), and combined therapy with Hévíz water and mud (**c**) (30 mins on every weekday by the end of the study). Data are shown as means±SEM of n = 6– 8 mice/ group; two-way ANOVA followed by Bonferroni's multiple comparison test.

The control weight bearing of the limbs was ca. 50-50% in every group. As a result of inflammation the values decrease moderately but in accordance with the values of the mechanonociceptive threshold. After one week, the drop of weight bearing on the treated limbs was the most pronounced and the pain was relieved by the end of the study. 6 days after CFA injection spontaneous weight bearing on the affected limbs decreased by 17% (from 51.81 + 1.71 to 42.78 + 2.47) in the tap water-treated group, but this was not significantly different compared to the Hévíz water-treated group (from 49.75 + 0.56 to 38.90 + 1.99, Figure 5(a)). Similar tendency and no significant difference could be detected in the other groups (Figures 5(b) and 5(c)).

### 3.4. Balneological Treatments Do Not Ameliorate MIA-Induced Structural Changes in the Bone

**Figure 6:**
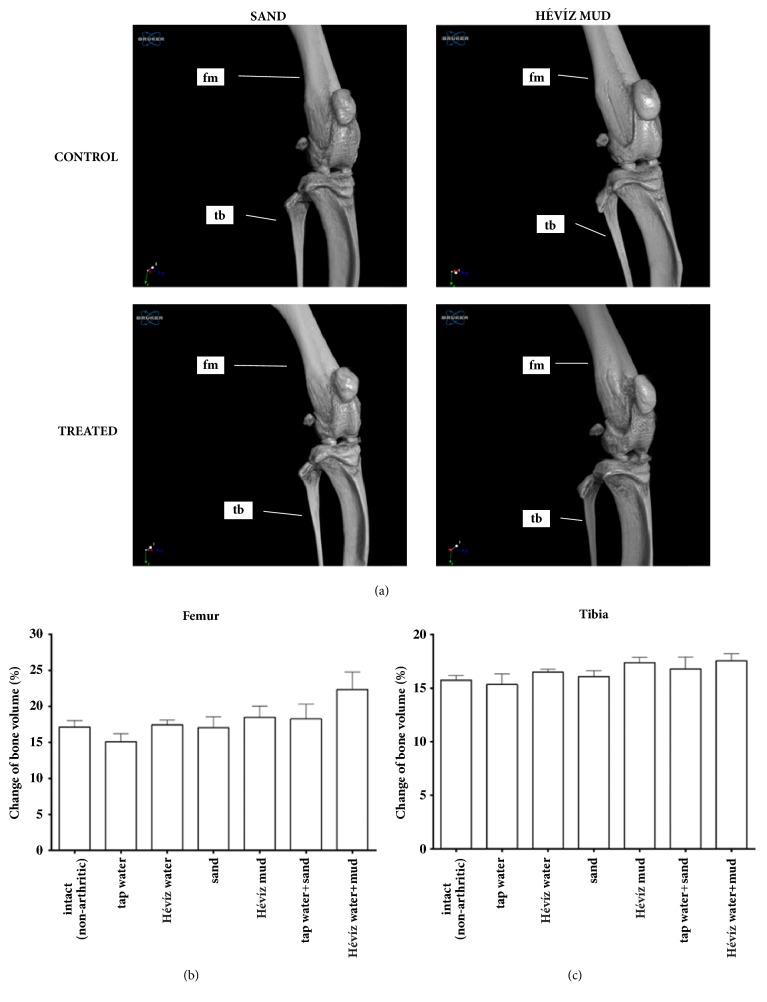
**Bone structural damage in the inflamed region in CFA-induced rheumatoid arthritis.** (a) Representative micro-CT images in intact state and on day 19. (b) Bone volume/total volume ratio in the femur, expressed as raw data and as percentage of the initial controls. (c) Bone volume/total volume ratio in the distal tibia, expressed as raw data and as percentage of the initial controls (n = 6/group, two-way ANOVA + Dunnett and Tukey posttests).

There were no considerable differences induced by MIA injection on the micro-CT scans between bone volume and total volume ration of mice treated with Hévíz water, mud, or their combination compared to the normal, intact condition or its respective controls. The basal bone mass both in the tibiotarsal and distal tibial regions was similar in each treated group at the end of the 19-day experiment (Figures 6(a), 6(b), and 6(c)).

### 3.5. Combined Treatment with Hévíz Water and Mud Significantly Decreases the Plasma Level of Interleukin-1*β* but Not the TNF-*α* Level in MIA-Induced Osteoarthritis Model

**Figure 7:**
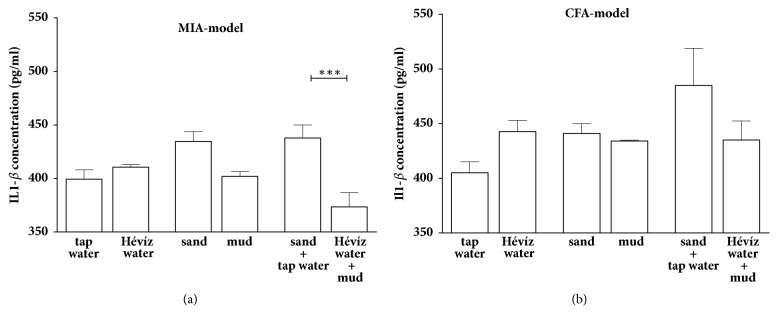
**Plasma concentrations of inflammatory cytokines interleukin 1-beta (IL1-** **β** **) in MIA-induced osteoarthritis (a) and in CFA-induced rheumatoid arthritis (b) models**. Each bar is represented as means±SEM of n=6-8 mice/group, *∗∗∗*p < 0.001 versus respective controls; one-way ANOVA followed by Bonferroni's multiple comparison test.

The serum concentrations of IL-1*β* were significantly attenuated by the combined treatment with Hévíz water and mud compared to the control mice in MIA-evoked osteoarthritis model (Figure 7(a)). IL-1*β* levels in the control and treated groups did not show any differences in the CFA-induced arthritis model (Figure 7(b)). Balneological interventions were not effective on TNF-*α* level either in MIA or CFA models (data not shown).

### 3.6. Balneological Treatments Did Not Alleviate Histopathological Changes Either in the Knee Joint in MIA-Induced Osteoarthritis, Or in the Tibiotarsal Joint in Rheumatoid Arthritis Model

**Figure 8:**
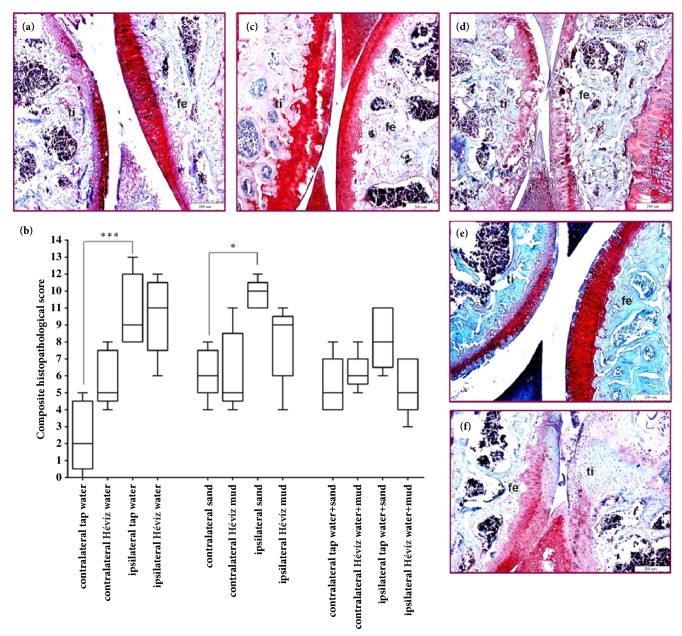
**Representative pictures and histopathological changes of the knee joints in MIA-induced osteoarthritis.** Representative slides stained with Safranin O of (**a**) an intact knee joint (ti: tibia, fe: femur), (**c**) tap water-treated, (**d**), tap water+sand-treated, (**e**) Hévíz water, and (**f**) Hévíz water+mud-treated mouse knee obtained on day 20 (200× magnification). (**b**) Semiquantitative scores obtained on the basis of structure, cartilage condition, area of erosion, synovial hyperplasia, synovial inflammatory cells infiltration, and osteophyte formation (due to van der Kraan et al. 1989). Box plots represent medians of composite scores for n = 6–9 mice/group; *∗*p< 0.05, *∗∗∗* p < 0.001 (versus respective contralateral joint); Kruskal-Wallis test followed by Dunn's posttest.

**Figure 9:**
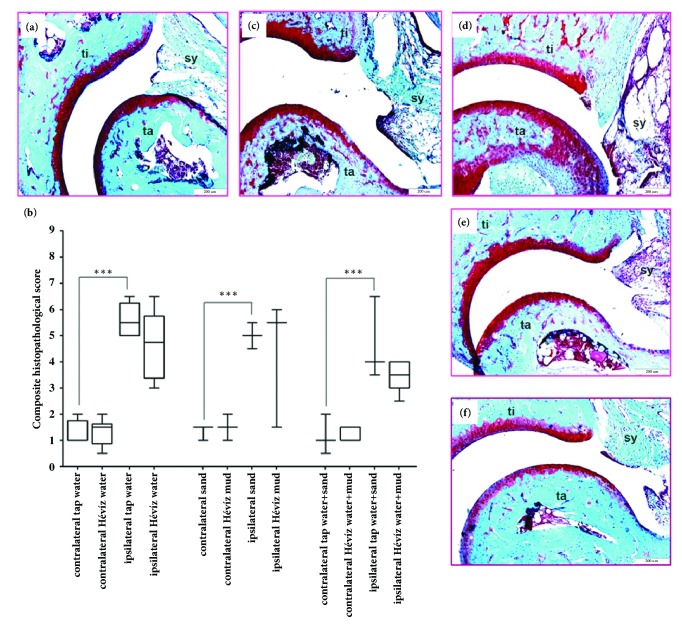
**Representative pictures and histopathological changes of the knee joints in CFA-induced rheumatoid arthritis.** Representative slides stained with Safranin O of (**a**) an intact tibiotarsal joint (ti: tibia, ta: tarsus, and sy: synovium), (**c**) tap water-treated, (**d**), tap water+sand-treated, (**e**) Hévíz water, and (**f**) Hévíz water+mud-treated mouse knee obtained on day 20 (100 × magnification).** (b)** Semiquantitative scores obtained on the basis of structure, cartilage condition, area of erosion, synovial hyperplasia, synovial inflammatory cells infiltration, and osteophyte formation (due to van der Kraan et al. 1989). Box plots represent medians of composite scores for n = 6–8 mice/group; *∗∗∗* p < 0.001 (versus respective contralateral joint); Kruskal-Wallis test followed by Dunn's posttest.

MIA or CFA injections induced a robust joint destruction in all groups. Characteristic chronic arthritic changes developed in the control mice by day 18 or 19, respectively, such as synovial hyperplasia with a moderate infiltration of mononuclear cells, fibroblast formation, and collagen deposition resulting in a composite arthritis score ca. 10-11 in MIA and 5-6 in CFA models. In balneotherapy-treated mice these changes were less pronounced and showed a tendency with smaller synovial swelling, reduced number of fibroblasts, and less collagen, but the difference was not significant. Histopathological scores of the three balneotherapy-administered groups did not differ significantly from their respective controls (Figures 8(b) and 9(b)).

## 4. Discussion

In the present study we developed and validated animal models for evaluation of anti-inflammatory and antinociceptive effects of the Hévíz thermal bath, mud, or their combined therapy. Furthermore we confirmed that balneotherapy is effective in murine model of degenerative arthritis. Hévíz mineral water and combined therapy significantly decreased the mechanical hyperalgesia, in MIA-induced osteoarthritis model. Knee oedema was also attenuated by the treatments but only the thermal water exerted significant action. Measurement of plasma concentration of inflammatory cytokines (IL-1*β*, TNF-*α*) revealed that combined treatment has substantially decreased the IL-1*β* level in MIA-model. However, balneotherapy did not influence mechanical hyperalgesia, paw oedema, and the spontaneous pain in CFA-induced rheumatoid arthritis model. Experimental data based on micro-CT analysis demonstrated that neither MIA nor CFA-treatment caused severe structural damage of the bones during the 19 day experimental period. Robust histopathological changes of the knee joints have been observed in both models, but balneotherapy itself proved to be ineffective.

Up to now, only limited number of controlled animal studies have been executed to reveal the effects of balneotherapy in different disease models of inflammation and pain [29–31]. Since pathological mechanisms of the human OA and RA are complex and can be activated by various endogenous and exogenous inflammatory and immune mediators, it is hardly possible to establish a perfect animal model. One of the main important issues is to find the sufficient process of mud treatment without anaesthesia. In a long term experiment (more than 10 days) it is not possible to anesthetize the experimental animals every day. The other capital problem is the most suitable application routine of balneotherapy in the experimental design. After 2 pretreatment processes, animals were easily habituated to the 35°C Hévíz or tap water bath. According to our personal experiences the appropriate mud therapy in the laboratory animals is very problematic especially in mice. Earlier studies have not given any instructions for technical details; therefore we have worked out an original special method. Avoiding the inhalation and ingestion of the mud, animals were enclosed into small tubes containing holes on the walls to allow fresh air exchange and perspiration [32]. The restraints exert stress reactions [33], but peloid administration might not be executed without restraint of the animals. Since control mice were also enclosed in these tubes during the treatment procedure, the influence of the stress-induced pain behaviour [33] on the experimental data could be eliminated. Since the 35°C tap water had not any effect on the inflammatory processes, thermal action of the water can be excluded. Therefore, chemical components of the mineral water are presumably important mediators of the therapeutic effect of balneotherapy. Besides solid compounds the water of Lake Hévíz is also rich in dissolved gas components, such as hydrogen sulphide (H_2_S). H_2_S is a small gas molecule, which can get into the body by diffusion through the skin or the airways during the bath treatment. H_2_S is known as an endogenous signalling molecule with multiple functions. Its involvement in the pathomechanisms of cardiovascular, nervous system, and inflammatory diseases is widely investigated [34–36]. H_2_S attenuates or enhances the inflammatory processes in animal models depending of the type of inflammation and H_2_S-concentration. H_2_S has several different actions [37]: it modifies proteins by S-sulfhydration [38], acts on ezymes (e.g., cytochrome c oxidase, kinases), and has influence on ion channels (e.g., L-type Ca^2+^ channels, chloride channels, transient receptor potential ankyrin 1 (TRPA1) ion channel, and K_ATP_ channel) [35, 36, 39–42]. Since we know that the capsaicin-sensitive sensory nerves densely innervate the joints and the TRPA1 receptors, expressed on these nerve terminals, a key molecule of nociception and local inflammatory responses [43], these ion channels can be a possible link between the mineral water and pain perception. Activation of TRPA1 through H_2_S can lead to somatostatin (sst) release from the nerve fibres [44] and it can exert antinociceptive effects acting on its G_i_-coupled sst_4_ receptor. Similar mechanism was suggested in the background of an inflammatory skin disease. Boros et al. [30] confirmed that the release of anti-inflammatory and analgesic neuropeptide somatostatin plays a crucial role in the mode of action of Harkány mineral water BT. The potential clinical relevance of our present experimental data is that H_2_S content of the sulfurous mineral waters can induce significant elevation of plasma somatostatin level and presumably that it is responsible for anti-inflammatory effects that can be observed in patients with psoriasis.

Another critical point is the transport of the minerals through the skin layers and their accumulation in the joints. Unfortunately, only few investigations have been conducted on these topics and there is a little knowledge about the specific actions of different mineral waters [45]. Nagy et al. previously reported an electrometric probe, developed to measure the H_2_S penetration through the skin of living experimental mouse [46]. This subcutaneous sensor could detect H_2_S penetration through the skin of patients treated with thermal bath or medicinal mud therapy. A recent paper has reported that exogenous H_2_S had an effect on the fibroblast-like synoviocytes which is one of the key players of OA pathogenesis. These cells can produce proinflammatory cytokines and matrix degrading enzymes. They found that H_2_S partially antagonized IL-1*β* stimulation via selective manipulation of the MAP kinase and the PI3K/Akt pathways which could open a new way to develop novel drugs for treatment of OA [47].

Nowadays, several human in vivo studies demonstrated the efficacy of BT [1–3, 9, 48, 49]. Randomized controlled clinical trials investigated OA of the knee [50, 51], hip [52], or shoulder [53].

Data concerning the effect of balneotherapy and mud therapy in CFA-induced rheumatoid arthritis model in rats demonstrate that sulfur bath and mud have an anti-inflammatory effect in the late phase of the arthritis [54–56] while it may exert a proinflammatory action in the acute phase of the inflammation [57]. In our murine model, Hévíz water and mud were ineffective in relieving the CFA-induced mechanical hyperalgesia, paw oedema, and the decrease of dynamic weight bearing, which are the main symptoms of rheumatoid arthritis in mice [58]. Our experimental data also support clinical observations in RA and OA patients, that balneo- and mud therapy exert significant beneficial effect in degenerative alterations but could be harmful in the acute phase of autoimmune-based inflammatory processes. [58, 59].

## 5. Conclusions

The present study provides valuable in vivo* experimental* data based on murine osteoarthritis and rheumatoid arthritis models to establish the effects of Hévíz mineral water and mud by modern methodological approach using functional tests and morphological analysis. Furthermore, our animal studies have provided experimental evidence for favourable effects of Hévíz water and mud that can be observed clinically in OA patients.

## Data Availability

No data were used to support this study.

## References

[1] Fioravanti A., Bacaro G., Giannitti C. (2015). One-year follow-up of mud-bath therapy in patients with bilateral knee osteoarthritis: a randomized, single-blind controlled trial. *International Journal of Biometerology*.

[2] Bender T., Bálint G., Prohászka Z., Géher P., Tefner I. K. (2014). Evidence-based hydro- and balneotherapy in Hungary--a systematic review and meta-analysis. *International Journal of Biometerology*.

[3] Kulisch A., Benko A., Bergmann A. (2014). Evaluation of the effect of Lake Heviz thermal mineral water in patients with osteoarthritis of the knee: a randomized, controlled, single-blind, follow-up study. *European Journal of Physical and Rehabilitation Medicine*.

[4] Krett G., Vágány V., Makk J. (2013). Phylogenetic diversity of bacterial communities inhabiting the sediment of Lake Hévíz-A comparison of cultivation and cloning. *Acta Microbiologica et Immunologica Hungarica*.

[5] Krett G., Nagymáté Z., Márialigeti K., Borsodi A. K. (2016). Seasonal and spatial changes of planktonic bacterial communities inhabiting the natural thermal Lake Hévíz, Hungary. *Acta Microbiologica et Immunologica Hungarica*.

[6] Gyarmati N., Kulisch A., Nemeth A. (2017). Evaluation of the Effect of Heviz Mud in Patients with Hand Osteoarthritis: A Randomized, Controlled, Single-Blind Follow-Up Study. *Israel Medical Association Journal*.

[7] Bailey E. E., Ference E. H., Alikhan A., Hession M. T., Armstrong A. W. (2012). Combination treatments for psoriasis: A systematic review and meta-analysis. *JAMA Dermatology*.

[8] Guidelli G. M., Tenti S., de Nobili E., Fioravanti A. (2012). Fibromyalgia syndrome and spa therapy: Myth or reality?. *Clinical Medicine Insights: Arthritis and Musculoskeletal Disorders*.

[9] Karagülle M., Kardeş S., Dişçi R., Karagülle M. Z. (2017). Spa therapy adjunct to pharmacotherapy is beneficial in rheumatoid arthritis: a crossover randomized controlled trial. *International Journal of Biometerology*.

[10] Hanzel A., Horvát K., Molics B. (2017). Clinical improvement of patients with osteoarthritis using thermal mineral water at Szigetvár Spa—results of a randomised double-blind controlled study. *International Journal of Biometerology*.

[11] Loeser R. F., Collins J. A., Diekman B. O. (2016). Ageing and the pathogenesis of osteoarthritis. *Nature Reviews Rheumatology*.

[12] Gálvez I., Torres-Piles S., Ortega-Rincón E. (2018). Balneotherapy, Immune System, and Stress Response: A Hormetic Strategy?. *International Journal of Molecular Sciences*.

[13] Harzy T., Ghani N., Akasbi N., Bono W., Nejjari C. (2009). Short- and long-term therapeutic effects of thermal mineral waters in knee osteoarthritis: a systematic review of randomized controlled trials. *Clinical Rheumatology*.

[14] Bathory G., Meretey K., Korda J. (1981). Kettős vak kísérlet a kiskunhalasi termálvíz terápiás hatásának mérésére rheumatoid arthritises betegeken. *Magy Reum*.

[15] Szucs L., Ratko I., Lesko T., Szoor I., Genti G., Balint G. (1989). Double-Blind Trial on the Effectiveness of the Puspokladany Thermal Water on Arthrosis of the Knee-Joints. *Journal of the Royal Society for the Promotion of Health*.

[16] Marker C. L., Pomonis J. D. (2012). The monosodium iodoacetate model of osteoarthritis pain in the rat. *Methods in Molecular Biology*.

[17] van der P. M., Kraan E. L., van de Putte L. B., van den Berg W. B. (1989). Development of osteoarthritic lesions in mice by “metabolic” and “mechanical” alterations in the knee joints. *The American Journal of Pathology*.

[18] Szabó Á., Helyes Z., Sándor K. (2005). Role of transient receptor potential vanilloid 1 receptors in adjuvant-induced chronic arthritis: in vivo study using gene-deficient mice. *The Journal of Pharmacology and Experimental Therapeutics*.

[19] Helyes Z., Szabó Á., Németh J. (2004). Antiinflammatory and analgesic effects of somatostatin released from capsaicin-sensitive sensory nerve terminals in a freund's adjuvant-induced chronic arthritis model in the rat. *Arthritis & Rheumatology*.

[20] Bölcskei K., Helyes Z., Szabó Á. (2005). Investigation of the role of TRPV1 receptors in acute and chronic nociceptive processes using gene-deficient mice. *PAIN*.

[21] Helyes Z., Pintér E., Németh J. (2006). Effects of the somatostatin receptor subtype 4 selective agonist J-2156 on sensory neuropeptide release and inflammatory reactions in rodents. *British Journal of Pharmacology*.

[22] Pertici V., Pin-Barre C., Felix M.-S., Laurin J., Brisswalter J., Decherchi P. (2014). A new method to assess weight-bearing distribution after central nervous system lesions in rats. *Behavioural Brain Research*.

[23] Hildebrand T., Laib A., Müller R., Dequeker J., Rüegsegger P. (1999). Direct three-dimensional morphometric analysis of human cancellous bone: microstructural data from spine, femur, iliac crest, and calcaneus. *Journal of Bone and Mineral Research*.

[24] Bouxsein M. L., Boyd S. K., Christiansen B. A., Guldberg R. E., Jepsen K. J., Müller R. (2010). Guidelines for assessment of bone microstructure in rodents using micro-computed tomography. *Journal of Bone and Mineral Research*.

[25] Botz B., Bölcskei K., Kereskai L. (2014). Differential regulatory role of pituitary adenylate cyclase-activating polypeptide in the serum-transfer arthritis model. *Arthritis & Rheumatology*.

[26] Borbély É., Botz B., Bölcskei K. (2015). Capsaicin-sensitive sensory nerves exert complex regulatory functions in the serum-transfer mouse model of autoimmune arthritis. *Brain, Behavior, and Immunity*.

[27] Borbély É., Hajna Z., Sándor K. (2013). Role of tachykinin 1 and 4 gene-derived neuropeptides and the neurokinin 1 receptor in adjuvant-induced chronic arthritis of the mouse. *PLoS ONE*.

[28] Mankin H. J., Dorfman H., Lippiello L., Zarins A. (1971). Biochemical and metabolic abnormalities in articular cartilage from osteo-arthritic human hips. II. Correlation of morphology with biochemical and metabolic data. *The Journal of Bone & Joint Surgery*.

[29] Zivna H., Maric L., Gradosova I. (2012). The effect of mud-bath therapy on bone status in rats during adjuvant subchronic arthritis. *Acta Medica*.

[30] Boros M., Kemény Á., Sebok B. (2013). Sulphurous medicinal waters increase somatostatin release: It is a possible mechanism of anti-inflammatory effect of balneotherapy in psoriasis. *European Journal of Integrative Medicine*.

[31] Bajgai J., Fadriquela A., Ara J. (2017). Balneotherapeutic effects of high mineral spring water on the atopic dermatitis-like inflammation in hairless mice via immunomodulation and redox balance. *BMC Complementary and Alternative Medicine*.

[32] Tékus V., Horváth Á., Hajna Z. (2016). Noxious heat threshold temperature and pronociceptive effects of allyl isothiocyanate (mustard oil) in TRPV1 or TRPA1 gene-deleted mice. *Life Sciences*.

[33] Scheich B., Vincze P., Szőke É. (2017). Chronic stress-induced mechanical hyperalgesia is controlled by capsaicin-sensitive neurones in the mouse. *European Journal of Pain*.

[34] Li L., Moore P. K. (2008). Putative biological roles of hydrogen sulfide in health and disease: a breath of not so fresh air?. *Trends in Pharmacological Sciences*.

[35] Burguera E. F., Meijide-Faílde R., Blanco F. J. (2017). Hydrogen sulfide and inflammatory joint diseases. *Current Drug Targets*.

[36] Hajna Z., Saghy E., Payrits M. (2016). Capsaicin-sensitive sensory nerves mediate the cellular and microvascular effects of H2S via TRPA1 receptor activation and neuropeptide release. *Journal of Molecular Neuroscience*.

[37] Li L., Rose P., Moore P. K. (2011). Hydrogen sulfide and cell signaling. *Annual Review of Pharmacology and Toxicology*.

[38] Mustafa A. K., Gadalla M. M., Sen N. (2009). H2S signals through protein S-Sulfhydration. *Science Signaling*.

[39] Malekova L., Krizanova O., Ondrias K. (2009). H(2)S and HS(-) donor NaHS inhibits intracellular chloride channels. *General Physiology and Biophysics*.

[40] Zhao W., Zhang J., Lu Y., Wang R. (2001). The vasorelaxant effect of H(2)S as a novel endogenous gaseous K_(ATP)_ channel opener. *EMBO Journal*.

[41] Streng T., Axelsson H. E., Hedlund P. (2008). Distribution and function of the hydrogen sulfide-sensitive TRPA1 ion channel in rat urinary bladder. *European Urology*.

[42] Pozsgai G., Payrits M., Saghy E. (2017). Analgesic effect of dimethyl trisulfide in mice is mediated by TRPA1 and sst4 receptors. *Nitric Oxide*.

[43] Horváth Á., Tékus V., Boros M. (2016). Transient receptor potential ankyrin 1 (TRPA1) receptor is involved in chronic arthritis: in vivo study using TRPA1-deficient mice. *Arthritis Research & Therapy*.

[44] Bátai I. Z., Horváth Á., Pintér E., Helyes Z., Pozsgai G. (2018). Role of transient receptor potential ankyrin 1 ion channel and somatostatin sst4 receptor in the antinociceptive and anti-inflammatory effects of sodium polysulfide and dimethyl trisulfide. *Frontiers in Endocrinology*.

[45] Fioravanti A., Karagülle M., Bender T., Karagülle M. Z. (2017). Balneotherapy in osteoarthritis: Facts, fiction and gaps in knowledge. *European Journal of Integrative Medicine*.

[46] Nagy L., Filotás D., Boros M., Pozsgai G., Pintér E., Nagy G. (2014). Amperometric cell for subcutaneous detection of hydrogen sulfide in anesthetized experimental animals. *Physiological Measurement*.

[47] Sieghart D., Liszt M., Wanivenhaus A. (2015). Hydrogen sulphide decreases IL-1*β*-induced activation of fibroblast-like synoviocytes from patients with osteoarthritis. *Journal of Cellular and Molecular Medicine*.

[48] Pascarelli N. A., Cheleschi S., Bacaro G., Guidelli G. M., Galeazzi M., Fioravanti A. (2016). Effect of mud-bath therapy on serum biomarkers in patients with knee osteoarthritis: Results from a randomized controlled trial. * Israel Medical Association Journal*.

[49] Giannitti C., De Palma A., Pascarelli N. A. (2017). Can balneotherapy modify microRNA expression levels in osteoarthritis? A comparative study in patients with knee osteoarthritis. *International Journal of Biometerology*.

[50] Branco M., Rego N. N., Silva P. H. (2016). Bath thermal waters in the treatment of knee osteoarthritis: a randomized controlled clinical trial. *European Journal of Physical and Rehabilitation Medicine*.

[51] Ciani O., Pascarelli N. A., Giannitti C. (2017). Mud-bath therapy in addition to usual care in bilateral knee osteoarthritis: an economic evaluation alongside a randomized controlled trial. *Arthritis Care & Research*.

[52] Kovács C., Bozsik Á., Pecze M. (2016). Effects of sulfur bath on hip osteoarthritis: a randomized, controlled, single-blind, follow-up trial: a pilot study. *International Journal of Biometerology*.

[53] Tefner I. K., Kovács C., Gaál R. (2015). The effect of balneotherapy on chronic shoulder pain. A randomized, controlled, single-blind follow-up trial. A pilot study. *Clinical Rheumatology*.

[54] Karagülle M., Tütüncü Z., Aslan O., Başak E., Mutlu A. (1996). Effects of thermal sulphur bath cure on adjuvant arthritic rats. *Physikalische Medizin, Rehabilitationsmedizin, Kurortmedizin*.

[55] Cozzi F., Carrara M., Sfriso P., Todesco S., Cima L. (2004). Anti-inflammatory effect of mud-bath applications on adjuvant arthritis in rats. *Clinical and Experimental Rheumatology*.

[56] Britschka Z. M. N., Teodoro W. R., Velosa A. P. P., De Mello S. B. V. (2007). The efficacy of Brazilian black mud treatment in chronic experimental arthritis. *Rheumatology International*.

[57] Kourilovitch M., Galarza-Maldonado C., Ortiz-Prado E. (2014). Diagnosis and classification of rheumatoid arthritis. *Journal of Autoimmunity*.

[58] Franke A., Reiner L., Resch K.-L. (2007). Long-term benefit of radon spa therapy in the rehabilitation of rheumatoid arthritis: a randomised, double-blinded trial. *Rheumatology International*.

[59] Sukenik S., Neumann L., Flusser D., Kleiner-Baumgarten A., Buskila D. (1995). Balneotherapy for rheumatoid arthritis at the Dead Sea. *Israel Journal of Medical Sciences *.

